# Cottonwood Clues in Fallon: Tree Rings Reflect Tungsten, Cobalt Exposure

**DOI:** 10.1289/ehp.115-a263b

**Published:** 2007-05

**Authors:** Valerie J. Brown

The cause of a childhood leukemia cluster in Fallon, Nevada (population 8,000) has mystified investigators since it was first discovered in 2000. Sixteen children have been diagnosed with acute lymphocytic leukemia and one with acute myelocytic leukemia. Because known risk factors such as ionizing radiation and prenatal exposure to volatile organic compounds do not explain most acute lymphocytic leukemia cases, researchers suspect other environmental exposures in Fallon. Now a tree ring study reveals elevated environmental tungsten and cobalt levels in Fallon compared to other towns in the area in the years just prior to the onset of the cluster **[*EHP* 115:715–719; Sheppard et al.]**.

Among Fallon’s potential sources of contamination are a tungsten carbide production facility, melon and alfalfa fields, and a naval air base jet fuel pipeline. The study team took core samples representing the years 1989 through 2002 from cottonwood trees around the Fallon processing plant and analyzed them for tungsten, cobalt (also used in tungsten carbide processing), and a range of other metals. For comparison, they also sampled trees in three nearby towns. In addition, the team tested trees in Sweet Home, Oregon, which also has a known local source of airborne tungsten, to test the dendrochemical technique independently.

Before 1992, median tungsten levels in Fallon tree rings differed little from those in the comparison Nevada towns, but rose thereafter to levels significantly higher than those in the other towns. Median cobalt levels in Fallon were higher than in surrounding towns but remained constant over the study period. Other trace metals did not increase consistently over time.

It is unknown whether tungsten causes cancer. The National Toxicology Program is currently assessing its disposition in rodents, with carcinogenicity studies planned. Cobalt has been associated with lung cancer, thyroid disorders, and lung disease, according to the CDC’s 2005 *Third National Report on Human Exposure to Environmental Chemicals*. The International Agency for Research on Cancer has classified the combination of tungsten carbide and cobalt as a probable human carcinogen.

By itself the tree-ring study does not establish a causal link between these elements and leukemia, but based on the temporal change in tungsten and the high level of cobalt found in the trees, further biomedical research is advisable.

## Figures and Tables

**Figure:**
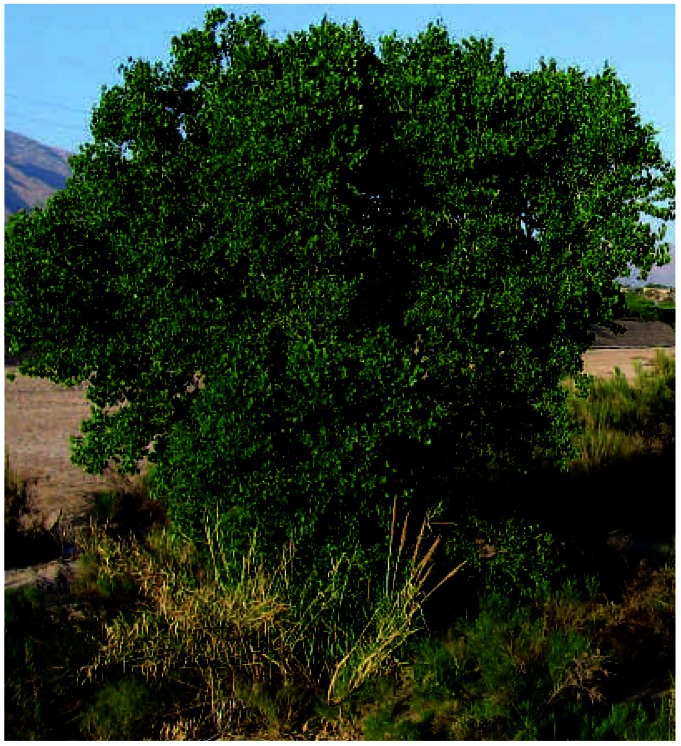
Branching out The cottonwoods around Fallon may add a new chapter to the story of the town’s leukemia cluster.

